# Fabrication of Chondroitin Sulfate–Copper/Zinc Complexes and Antibacterial Activity Involving Hydrogel Application in Infected Wound Healing

**DOI:** 10.3390/gels12070633

**Published:** 2026-07-15

**Authors:** Qingshan Shen, Jiarui Wu, Jiawen Li, Yujie Dong, Yang Liu, Lei Zhao, Huan Zhan, Yanli Ma

**Affiliations:** 1 Henan Key Laboratory of Zhang Zhongjing Formulae and Herbs for Immunoregulation, Zhang Zhongjing College of Chinese Medicine, Nanyang Institute of Technology, Changjiang Road 80, Nanyang 473004, China; 2 School of Food and Health, Henan College of Classical Chinese Medicine, Hansheng Road 1222, Nanyang 473000, China

**Keywords:** chondroitin sulfate–metal complex, antibacterial activity, hydrogel, wound healing

## Abstract

The escalating prevalence of bacterial infections has intensified the search for innovative antimicrobial strategies, particularly for infected wound management. Chondroitin sulfate (CS), a naturally occurring glycosaminoglycan with established biocompatibility, presents an attractive scaffold for developing metal ion-functionalized biomaterials. This study reports the fabrication of chondroitin sulfate–copper complex (CSCu) and chondroitin sulfate–zinc complex (CSZn) through an ion exchange method, wherein Cu^2+^ and Zn^2+^ ions bind to the groups of carboxylate, sulfate, or N-acetyl from the CS backbone. The resulting complexes exhibited copper or zinc loading capacities of about 6.6% and demonstrated potent antibacterial activity against *E. coli* and *S. aureus*. The integration of CSCu or CSZn with sodium alginate yielded a hydrogel system with a higher apparent viscosity, possessing injectability and spreadability on the skin surface and a porous three-dimensional internal structure conducive to wound healing applications. In a murine model of *S. aureus*-infected full-thickness wounds, topical application of CSCu and CSZn hydrogels substantially accelerated wound closure, achieving 97.46% and 98.11% healing, respectively, by day 10. Additionally, treatment with CSCu or CSZn hydrogels significantly attenuated systemic inflammatory responses, as reflected in lowered serum TNF-α, IL-1β, and IL-6 alongside increased IL-10. Histological evaluation confirmed enhanced re-epithelialization and stratum spinosum formation in treated wounds. These findings establish CSCu and CSZn as a promising bioactive agent for addressing bacterial wound infections through a dual mechanism of direct antibacterial action and immunomodulatory effects, offering a valuable alternative to conventional antibiotic therapies.

## 1. Introduction

The global burden of chronic and infected wounds represents a significant clinical challenge, with profound implications for patient quality of life and healthcare economics. Common chronic wounds encompass venous ulcers and arterial ulcers. Skin serves as the primary physical barrier essential for maintaining internal homeostasis, and upon injury, the body mobilizes sophisticated reparative pathways to regain tissue normality [1]. Nevertheless, the healing process can be derailed by unfavorable conditions, such as infection or hyperglycemia, ultimately resulting in chronic wounds [2]. *Staphylococcus aureus* (*S. aureus*), identified as the predominant pathogen in wound contamination worldwide, is a principal factor complicating wound healing through bacterial infection [3,4]. The remarkable adaptability of *S. aureus* and its propensity to acquire antimicrobial resistance genes have rendered conventional antibiotic therapies increasingly ineffective, necessitating the development of alternative antimicrobial strategies [5].

Metal-based antimicrobial agents have garnered considerable attention as promising candidates to address the escalating crisis of antibiotic resistance. Notably, copper ions (Cu^2+^) and zinc ions (Zn^2+^) possess strong, broad-spectrum bactericidal properties, effective against both Gram-positive and Gram-negative strains [6]. The generation of reactive oxygen species (ROS) via the Fenton reaction is central to Cu’s antibacterial mechanism, as these ROS inflict oxidative damage on DNA, proteins, and membrane lipids [7,8]. Furthermore, Cu toxicity is specifically linked to the inactivation of solvent-exposed iron–sulfur clusters in key metabolic enzymes, such as isopropylmalate isomerase and fumarase, which cripples core metabolic pathways including the biosynthesis of branched-chain amino acids and the TCA (tricarboxylic acid) cycle. Similarly, an excess of Zn ions disrupts bacterial metal homeostasis. High concentrations of Zn can outcompete essential cofactors, like manganese, for binding sites on critical proteins, leading to mismetallation of enzymes, such as superoxide dismutase, and impairing the bacterial oxidative stress defense [9,10]. Zinc is also known to target and destabilize Fe-S clusters in dehydratases, further compromising bacterial metabolism and fitness [11,12]. These distinctive mechanisms position copper and zinc ions as attractive alternatives to conventional antibiotics that bacteria have progressively evolved to resist. However, the direct application of free copper or zinc ions is constrained by several limitations, including rapid clearance, potential cytotoxicity to mammalian cells at elevated concentrations, and challenges in achieving sustained local delivery at therapeutic sites [13,14]. To circumvent these issues, substantial research efforts have focused on incorporating copper or zinc ions into biocompatible carrier matrices, including nanoparticles, hydrogels, and polysaccharide complexes [15,16,17].

As a sulfated glycosaminoglycan richly found in cartilage and connective tissues, chondroitin sulfate (CS) offers an outstanding scaffold platform for the design of metal ion-functionalized biomaterials. The anionic polysaccharide CS, built from repeating disaccharides of glucuronic acid and N-acetylgalactosamine, possesses plentiful carboxylate and sulfate groups that serve as binding sites for metal cations [18]. This inherent chelating capacity enables the formation of stable polysaccharide–metal complexes, wherein the biological activities of both components may be synergistically enhanced. The ion exchange methodology as an effective strategy for developing polysaccharide–metal complexes has been reported. Previous investigations have demonstrated that CS can complex with various metal ions, including calcium and iron [19,20]. In particular, chondroitin sulfate–iron complex (CSFe) has exhibited antibacterial activity and efficacy in promoting infected wound healing. However, the minimum inhibitory concentration (MIC) of CSFe against *Escherichia coli* (*E. coli*) and *S. aureus* was 10 mg/mL, while its Fe loading was only 2.06%.

**Figure 1:**
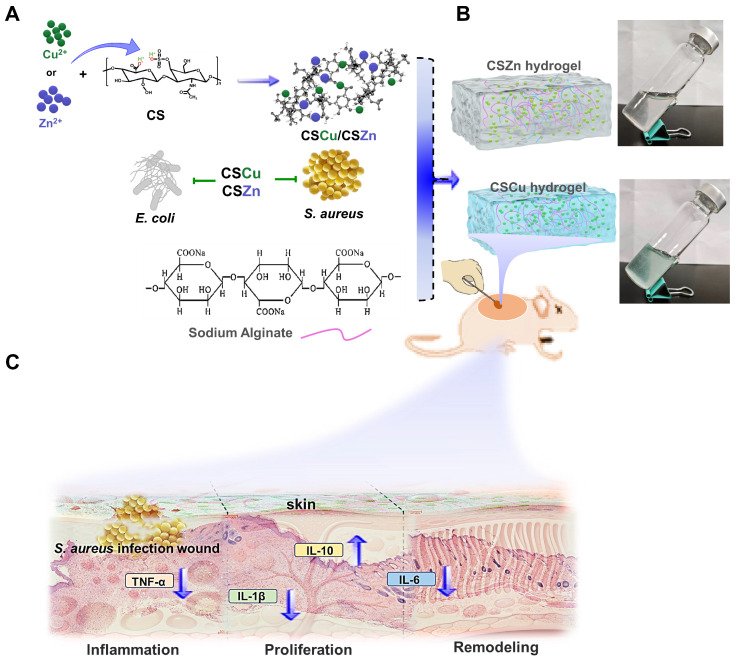
Polysaccharide–metal complex (CSCu or CSZn) hydrogel for treatment of *S. aureus*-infected wound efficacy. (**A**) Preparation of CSCu or CSZn complex, involving antibacterial activity, and a schematic illustration showing the hydrogel composition and formulation design. (**B**) The static photographs of CSCu and CSZn hydrogels at rest. (**C**) The CSCu or CSZn hydrogel for *S. aureus*-infected wound efficacy.

Building upon these precedents, the present study aimed to fabricate novel chondroitin sulfate–copper complex (CSCu) and chondroitin sulfate–zinc complex (CSZn) and comprehensively evaluate their physicochemical characteristics, antibacterial properties, and therapeutic potential for infected wound management (Figure 1). The specific objectives encompassed: (1) fabrication and structural characterization of CSCu and CSZn using ion exchange methods and spectroscopic and chromatographic techniques; (2) assessment of antibacterial activity against *E. coli* and *S. aureus*; (3) formulation and characterization of CSCu- or CSZn-loaded hydrogel systems; and (4) evaluation of wound healing efficacy in an in vivo model of *S. aureus*-infected wounds. This research contributes to the expanding repertoire of metal–polysaccharide biomaterials and offers insights into the development of innovative therapeutic strategies for combating antibiotic-resistant wound infections.

## 2. Results and Discussion

### 2.1. Fabrication and Characteristics of CSCu or CSZn

**Figure 2:**
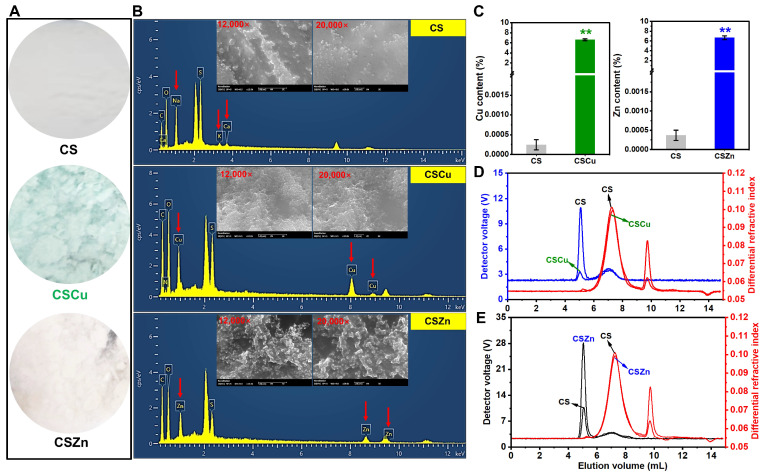
Fabrication and characteristics of CSCu and CSZn. (**A**) CSCu, CSZn, and CS sample powder; (**B**) SEM images and element mapping of CSCu, CSZn, and CS. SEM micrographs with EDS elemental maps; red arrows indicate elements that disappeared (Na, K, Ca) or newly emerged (Cu, Zn) signals. (**C**) Cu or Zn content (%) in CSCu, CSZn, and CS. The asterisk (**) indicates significant difference (*p* < 0.01). Data are presented as mean ± SD (*n* = 3). Statistical significance was determined by Student’s *t*-test. Chromatographic signal profiles of CSCu (**D**) and CSZn (**E**), together with CS, were acquired using multi-angle laser light scattering and refractive index detectors for Mw measurement.

CSCu or CSZn was successfully synthesized as a light blue and white powder following ion exchange reaction, dialysis purification, and lyophilization. The characteristic blue coloration, absent in native CS, provided preliminary visual confirmation of copper incorporation (Figure 2A). Except for some obvious layered structures on the CSCu sample and a blocky protrusion on the CSZn sample, SEM imaging revealed similar surface morphologies between CSCu, CSZn, and native CS at equivalent magnifications; however, elemental mapping analysis demonstrated distinct differences in elemental composition (Figure 2B). Notably, elements originally present in CS (Na, K, and Ca) were substantially diminished in CSCu and CSZn, while copper and zinc signals were clearly detected, indicating successful ion exchange. Quantitative ICP-MS analysis confirmed a copper content of 6.60% in CSCu and a zinc content of 6.66% in CSZn, which was significantly higher (*p* < 0.01) than that of native CS (Figure 2C). The Mw of CSCu (3.75 × 10^5^ Da) and CSZn (7.05 × 10^5^ Da) substantially exceeded that of native CS, as shown from the chromatograms determined by GPC-MALLS (Figure 2D,E).

CS, as an anionic polysaccharide, possesses abundant carboxyl and sulfate functional groups, together with N-acetyl groups, that serve as coordination sites for metal cations [18]. Under physiological conditions, CS typically exists as sodium, potassium, or calcium salts, with other divalent metal ions such as Mg^2+^, Sr^2+^, or Fe^3+^ interacting much more strongly with CS to form stable polysaccharide–metal complexes by ion exchange reaction [19,21,22]. The binding mechanisms involve electrostatic interactions, charge density effects, and ionic strength considerations [18]. The marked increase in molecular weight suggests that copper coordination induces intermolecular crosslinking or aggregation of CS chains, a phenomenon consistent with previous reports on polysaccharide–metal complex formation [19,20]. The substantially higher copper or zinc content achieved in CSCu (6.60%) or in CSZn (6.66%) compared to analogous chondroitin sulfate–iron complexes (2.06% Fe) [19], which indicates preferential binding affinity of CS for Cu^2+^ and Zn^2+^ over Fe^3+^.

### 2.2. Structural Properties of CSCu or CSZn

**Figure 3:**
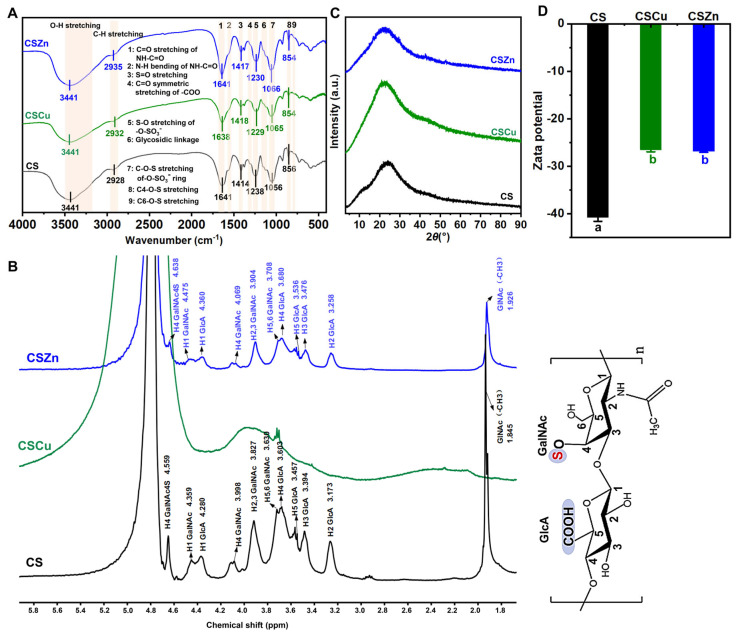
Structural properties of CSCu, CSZn, and CS. FTIR (**A**); ^1^H–NMR (**B**) spectra of CSCu, CSZn, and CS; XRD spectra (**C**) and zeta potential (**D**) of CSCu, CSZn, and CS. Data are presented as mean ± SD (*n* = 3). Different letters indicate significant difference (*p* < 0.01, one-way ANOVA, Duncan’s test).

FTIR spectroscopy provided insights into the coordination interactions between Cu^2+^, Zn^2+^ and CS functional groups (Figure 3A). While the overall spectra of CSCu, CSZn, and CS were broadly similar, some differences were observed in the 2000–1000 cm^−1^ region. The strong and wide band near 3500–3200 cm^−1^ and around 2932 cm^−1^ corresponds to O–H stretching and C-H vibrations, respectively [23], which is observed at 3441 cm^−1^ and 2828–2935 cm^−1^ in the spectra of CSCu, CSZn, and CS. The peaks near 1642 cm^−1^ were assigned to C=O stretching vibrations of NH-C=O [24]. Here, the absorption peak at 1638 cm^−1^ of CSCu was observed, while those of CSZn and CS appeared at 1641 cm^−1^. This means that the Cu^2+^, instead of Zn^2+^, binding site occurs at N-acetyl groups of CS, and this binding pattern is probably similar to that of the chondroitin sulfate–iron complex [19]. As reported by reference [25], the absorption band centered at 1400 cm^−1^ is a signature vibration for S=O stretching. Prior research also identifies the asymmetric -SO_2_ stretching peak of CS at 1255 cm^−1^ [20]. Our current measurements display shifted vibrational bands for CSCu and CSZn: the S=O stretching vibrations occur near 1418 cm^−1^ and 1417 cm^−1^, and asymmetric -SO_2_ stretching signals are detected at 1229 cm^−1^ and 1230 cm^−1^, respectively. Compared with the 1414 cm^−1^ and 1238 cm^−1^ peaks of unmodified CS, such spectral variations may arise from the interference of Cu^2+^ or Zn^2+^ cations on S=O and -SO_2_ stretching modes. These spectral perturbations suggest that Cu^2+^ coordination occurs at both N-acetyl and sulfate functional groups of CS, while Zn^2+^ mainly binds to sulfate groups.

The characteristic proton chemical shifts in the ^1^H-NMR spectra of CS fall within the range of 3–5 ppm, consistent with our earlier published work [20]. Overall, the ^1^H–NMR spectra of CS and CSZn are similar, but that of CSCu exhibits significant line broadening and suppression of proton signals, which is due to the paramagnetic properties of Cu^2+^ (Figure 3B). The ^1^H NMR spectrum of CSCu exhibited significant line broadening and complete suppression of proton signals due to the paramagnetic nature of the Cu^2+^ ions (which possess unpaired electrons). In fact, only the solvent (deuterium oxide) signal was readily observable in the CSCu spectrum, a phenomenon analogous to that previously reported for paramagnetic Fe^3+^ in CSFe [19]. This indicates that conventional ^1^H–NMR spectroscopy is unsuitable for structural analysis of polysaccharide complexes containing paramagnetic metal ions, such as Cu^2+^ and Fe^3+^, necessitating an alternative analytical approach, such as electron paramagnetic resonance for definitive coordination analysis. Compared with the CS spectrum between 3.4 and 4.8 ppm, the signals of GlcA and GalNAc protons in the CSZn spectrum changed, as shown in Table 1. The changes in protons in the CSZn spectrum are probably induced by the binding of Zn and CS. Especially, the signal attributed to H5 near the carboxyl group of GlcA in the CS spectrum is 3.457 ppm, while that in CSZn is 3.536 ppm. And, H1 (4.475 ppm) and H2/H3 (3.904 ppm) signals near the N-acetyl group of GalNAc in CSZn are also different from CS (H1: 4.359 ppm, H2/H3: 3.827 ppm). This means that the binding sites of Zn^2+^ and CS are the N-acetyl groups and carboxyl groups.

XRD patterns of CSCu, CSZn, and native CS exhibited broad amorphous peaks in the 2*θ* range of 20–40°, without sharp diffraction peaks characteristic of crystalline structures (Figure 3C). This observation indicates that Cu^2+^ and Zn^2+^ incorporation does not induce crystallization of CS, and CSCu and CSZn complexes retain the amorphous nature of the parent polysaccharide. The maintenance of an amorphous structure is advantageous for biomedical applications, as amorphous materials typically exhibit enhanced solubility and bioavailability [26] compared to crystalline counterparts.

Zeta potential measurements revealed that native CS exhibited a highly negative surface charge (–40.77 ± 0.80 mV), consistent with its anionic polyelectrolyte character (Figure 3D). Following copper coordination, the zeta potential of CSCu decreased to −26.53 ± 0.40 mV (*p* <0.01). This significant reduction in negative surface charge arises from charge neutralization upon Cu^2+^ binding to anionic carboxyl and sulfate groups. Generally, lower absolute zeta potential values correlate with reduced colloidal stability and increased propensity for aggregation, explaining the elevated molecular weight observed for CSCu and providing further evidence for complex formation rather than a simple physical mixture.

### 2.3. Antibacterial Activity of CSCu or CSZn

**Figure 4:**
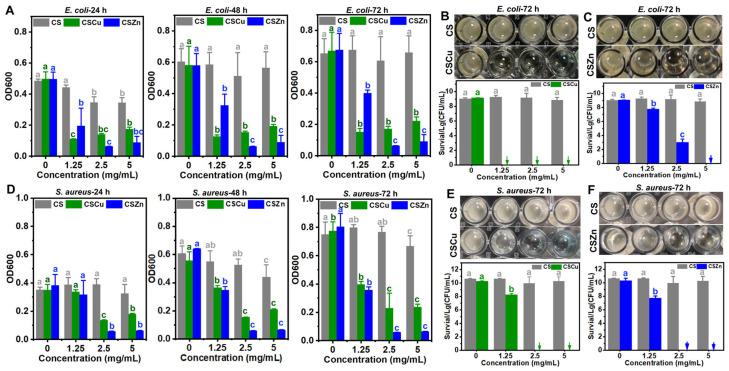
Comparative assessment of the antibacterial effects of CSCu, CSZn, and CS against *E. coli* and *S. aureus*. Bacterial growth (OD600) of *E. coli* (**A**–**C**) and *S. aureus* (**D**–**F**) was monitored over 24–72 h at 37 °C following exposure to varying concentrations (0–5 mg/mL) of CS, CSCu, or CSZn. All values are expressed as mean ± SD (*n* = 3), with significance determined via one-way ANOVA followed by Duncan’s multiple-range test (different letters within the same color denote *p* < 0.05). The olive green or blue arrows indicate that no bacterial colonies were observed on the agar plates.

The antibacterial capacities of CS, CSCu, and CSZn against *E. coli* and *S. aureus* were tested. As displayed in Figure 4A, CS failed to restrain the reproduction of *E. coli* at all measured concentrations (0–5 mg/mL) during 48–72 h cultivation. Notably, 1.25 mg/mL CSCu induced statistically significant (*p* < 0.05) suppression of *E. coli* growth and rendered the LB medium transparent. Analogous antibacterial performance was obtained with CSZn at a concentration of 2.5 mg/mL. Additionally, no CFU of *E. coli* treated with CSCu (over 1.25 mg/mL) (Figure 4B) and CSZn (over 2.5 mg/mL, even 5 mg/mL) (Figure 4C) for 72 h was observed on LB agar plate. This means that CSCu is able to kill *E. coli* when the final concentration reaches 1.25 mg/mL, and 5 mg/mL of CSZn is needed.

In terms of *S. aureus* (Figure 4D), exposure to CS at 5 mg/mL (based on OD600 measurements) appeared to restrain bacterial growth over 48 and 72 h of incubation; however, the TSB medium remained visually turbid. In contrast, at equivalent concentrations (≥1.25 mg/mL), both CSCu and CSZn elicited marked suppression of *S. aureus* proliferation (*p* < 0.05), with the TSB medium becoming visibly clear at 2.5 and 5 mg/mL. Moreover, no colony-forming units (CFUs) were detected on TSB agar plates at these two concentrations (Figure 4D–F), indicating that CSCu and CSZn, when applied at ≥2.5 mg/mL, exert bactericidal effects against *S. aureus*. Accordingly, CSCu exhibited MIC values of 1.25 mg/mL against *E. coli* and 2.5 mg/mL against *S. aureus*, while CSZn showed a uniform MIC of 2.5 mg/mL for both strains.

The antimicrobial activity of pristine CS, particularly at low concentrations, remains generally inadequate. For example, even when applied at 50 mg/mL, CS exhibits negligible inhibitory effects against *E. coli* [27]. In comparison, commercial CS demands MICs as high as 360 mg/mL and 420 mg/mL to suppress *E. coli* and *S. aureus*, respectively [28]. Notably, the polysaccharide–metal complex CSCu displays potent antibacterial, and even bactericidal, activity against both *E. coli* and *S. aureus*, with corresponding MIC values of 1.25 mg/mL and 2.5 mg/mL. Furthermore, at both MIC and 2× MIC levels, CSCu and CSZn are capable of effectively eradicating viable cells of these two pathogens, while unmodified CS barely interferes with their growth. Collectively, these observations imply that the robust antibacterial or bactericidal efficacy of CSCu and CSZn is largely ascribed to the chelation/introduction of Cu^2+^ and Zn^2+^ ions. Compared with the antibacterial CSFe, which needs 10 mg/mL [19], CSCu and CSZn exhibit stronger antibacterial ability. Research indicates that overloading with metal ions, such as Fe^3+^, Cu^2+^, and Zn^2+^, can trigger cell death named ferroptosis, cuproptosis, or disulfidptosis [29,30]. However, this type of death model usually occurs in mammalian cells. It was reported that Cu kills bacteria by damaging Fe-S clusters and key metabolic enzymes (e.g., GAPDH, IPMI, GOGAT), and the bacteria resist via small molecules (glutathione, amino acids) and metabolic bypasses [6]. And, Zn can cause mismetalization of superoxide dismutase and damage bacterial oxidative stress defense [9,10]. Here, the bactericidal potential of CSCu and CSZn against the two tested bacterial strains is presumably due to this metal-associated death mechanism, highlighting their potential utility in the development of novel antibacterial materials.

The antibacterial mechanism of CSCu and CSZn can be understood through a multi-step process: ion release, ROS generation, and bacterial damage. Upon application, Cu^2+^ and Zn^2+^ are gradually released from the CS coordination sites via ion exchange with physiological cations. Once internalized by bacteria, Cu^2+^ undergoes a Fenton-like reaction with endogenous H_2_O_2_, generating highly cytotoxic ROS [31]. Through the cyclic Cu^2+^/Cu^+^ redox reaction, ROS generation is amplified, culminating in peroxidative damage to lipids, loss of membrane integrity, oxidative protein modification, and genomic DNA injury. Recent evidence indicates that Cu^2+^ can induce ferroptosis-like death in *S. aureus* by increasing intracellular Fe^2+^, elevating ROS and lipid peroxidation, and triggering the intracellular Fenton reaction [32]. Zn^2+^, in turn, induces oxidative stress through ROS generation and disrupts bacterial membrane integrity [33]. Beyond ROS-mediated damage, Cu^+^ directly penetrates bacterial membranes and inactivates solvent-exposed iron–sulfur clusters in key metabolic enzymes, crippling core pathways, such as the TCA cycle [34], while Zn^2+^ causes mismetallation of bacterial enzymes (e.g., superoxide dismutase), impairing oxidative stress defense [9,10]. Importantly, the CS carrier enables sustained release of these metal ions, ensuring prolonged antibacterial activity while minimizing systemic toxicity. This multi-targeted mechanism, involving membrane disruption, oxidative damage, and metabolic interference, makes it inherently difficult for bacteria to develop resistance, positioning CSCu and CSZn as promising alternatives to conventional antibiotics.

### 2.4. Characteristics of CSCu or CSZn Hydrogels

**Figure 5:**
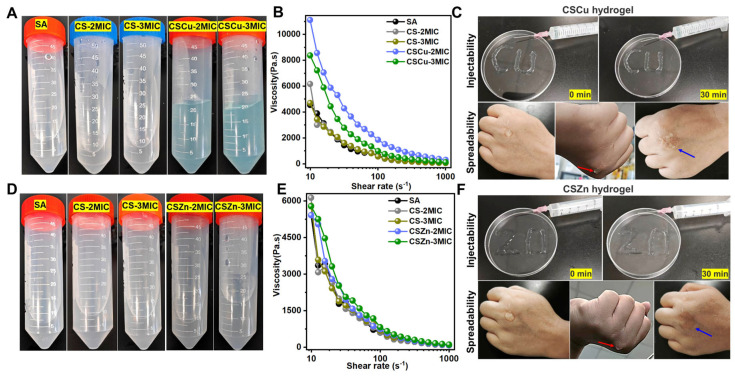
Characteristics of CSCu and CSZn hydrogels. The status (**A**,**D**) of SA, CS, CSCu, and CSZn hydrogels when inclined at approximately 20°. Viscosity (**B**,**E**) of the SA, CS, CSCu, and CSZn hydrogels at 2MIC and 3MIC. The injectability and spreadability of CSCu (**C**) and CSZn (**F**) hydrogels were assessed by syringe and assessed on the skin surface, respectively. Red arrows indicate that the hydrogels, after movement, do not fall off even when bent. Blue arrows indicate the spreadability of hydrogels on the skin.

The incorporation of CSCu or CSZn into SA matrices yielded hydrogel systems with distinct physical properties compared to native CS or pure SA hydrogels (Figure 5). SA, an anionic polysaccharide composed of mannuronic and guluronic acid residues, has been extensively investigated for hydrogel formation due to its capacity for ionotropic gelation with multivalent cations or through covalent crosslinking [35]. SA-based hydrogels offer excellent biocompatibility, effective exudate absorption, and favorable moisture retention, making them well-suited for wound dressing applications.

Visual assessment of hydrogel flowability at a 20° inclination angle revealed that pure SA and native CS hydrogels exhibited greater flowability compared to CSCu hydrogels, which, especially at 2MIC of CSCu, mainly maintained a stable jelly-like consistency (Figure 5A). Interestingly, the flowability of CSCu hydrogel at 3MIC seemed to be greater than 2MIC of CSCu hydrogel. This means that for CSCu, the higher the concentration, the thicker the hydrogel is not necessarily. These results were confirmed by the apparent viscosity of the hydrogels. 2MIC of CSCu hydrogel possessed the highest apparent viscosity among corresponding 3MIC of CSCu, CS, or SA formulations across the entire shear rate range tested (1–1000 s^−1^) (Figure 5B). All hydrogel formulations exhibited shear-thinning behavior, with viscosity decreasing progressively as shear rate increased, ultimately reaching dynamic equilibrium. This shear-thinning characteristic is advantageous for wound dressing applications, as it facilitates application and spreading under gentle pressure while maintaining structural integrity at rest. For CSZn hydrogel, higher concentration corresponded to lower flowability (Figure 5D) and greater apparent viscosity (Figure 5E) of the hydrogel. This means that CSCu has a stronger crosslinking capacity than that of CSZn. In other words, copper ions with SA have a stronger crosslinking effect than zinc ions. The viscosity values of CSCu and CSZn hydrogels at 2MIC and 3MIC concentrations are comparable to or higher than those of SA-based hydrogels for wound dressing applications, which typically exhibit viscosities at low shear rates. The shear-thinning behavior observed across all formulations is characteristic of physically crosslinked polysaccharide hydrogels and is advantageous for topical wound care, as it allows easy spreading under gentle shear while maintaining structural integrity at rest. Notably, the higher apparent viscosity of 2MIC CSCu hydrogel compared to 3MIC CSCu suggests that excessive crosslinker concentration may disrupt the optimal network structure, a phenomenon that warrants further investigation.

Notably, CSCu or CSZn hydrogels at 3MIC concentrations demonstrated excellent injectability through standard syringes, maintaining structural integrity upon extrusion. Additionally, these hydrogels exhibited favorable spreadability when applied to human skin surfaces (Figure 5C,F), suggesting practical applicability for topical wound treatment. The enhanced flowability and viscosity properties of CSCu or CSZn hydrogels likely result from additional physical crosslinking mediated by copper ions and zinc ions, which can interact with both carboxyl groups of SA and remaining uncoordinated sites on CSCu or CSZn, thereby reinforcing the hydrogel network structure.

The practical adhesion performance of CSCu and CSZn hydrogels under dynamic mechanical stress was further evaluated by applying the hydrogels to human hand skin and knuckle joints, followed by repeated flexion–extension movements (Supplementary Videos S1 and S2). The hydrogels remained firmly attached to the skin surface throughout the movement cycles, with no visible detachment, cracking, or loss of gel integrity. This qualitative assessment, together with the shear-thinning rheological behavior (Figure 5B,E) and spreadability of CSCu and CSZn hydrogels shown in Figure 5C,F, confirms the suitability of CSCu or CSZn hydrogels for topical application on mobile wound sites. Quantitative adhesion strength measurements, compared with commercial wound dressings, will be systematically addressed in our future work.

### 2.5. Structure of CSCu or CSZn Hydrogels

**Figure 6:**
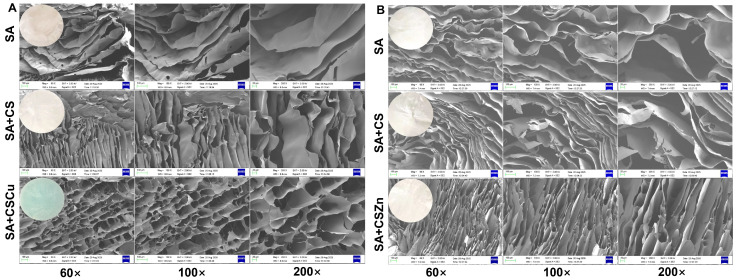
The internal morphological structure of hydrogels. The surface (the insert images) and interior morphology of freeze-dried hydrogels of CSCu (**A**) and CSZn (**B**), together with SA, respectively, as observed at 60×, 100×, and 200× magnifications.

SEM examination of freeze-dried hydrogels provided insights into the internal architecture of CSCu- or CSZn-loaded matrices (Figure 6). While surface morphologies (the insert graphs) of SA, CS, CSCu, and CSZn hydrogels appeared superficially similar at low magnification, tactile assessment revealed that CSCu and CSZn hydrogels were substantially firmer than their CS or SA counterparts at equivalent concentrations. This increased mechanical rigidity aligns with the enhanced viscosity observed in rheological studies. Cross-sectional imaging of lyophilized hydrogels revealed striking differences in internal pore architecture (Figure 6A,B). CSCu or CSZn hydrogels exhibited a regular, highly porous three-dimensional network structure characterized by smaller, more uniform pores and greater structural compactness compared to the relatively loose, irregular networks of SA and CS hydrogels. This indicates that copper and zinc can change the internal structure of the hydrogel. It is well documented that an ordered porous architecture is beneficial for wound healing, since scaffolds with high porosity and interconnected pore networks are known to promote cell adhesion, proliferation, and migration, while also facilitating gas exchange and nutrient supply [36,37]. Accordingly, CSCu or CSZn hydrogels may serve as potential candidates for tissue engineering scaffold applications.

The enhanced structural integrity and reduced pore size of CSCu and CSZn hydrogels likely arise from copper- and zinc-mediated crosslinking between alginate chains and CSCu or CSZn molecules. This denser network, characterized by enhanced interactions between water molecules and polymer chains, accounts for the elevated viscosity and diminished flowability observed in CSCu and CSZn hydrogels. These structural attributes—high porosity, mechanical stability, and injectability—collectively position CSCu or CSZn hydrogels as promising candidates for advanced wound dressing applications.

While the present study focused on viscosity, injectability, and morphological characterization of the hydrogels, we acknowledge that quantitative swelling ratio, degradation behavior, and mechanical properties were not systematically evaluated. The porous three-dimensional network observed by SEM suggests that the hydrogels possess a good water uptake capacity, which is important for absorbing wound exudate and maintaining a moist healing environment. Additionally, the shear-thinning rheological behavior and the compact porous structure imply some mechanical integrity for wound dressing applications. However, we recognize that direct measurements of swelling kinetics, in vitro degradation profiles, and compressive modulus would provide a more rigorous evaluation. These aspects will be systematically addressed in our future investigations to fully establish the performance profile of CSCu or CSZn hydrogels.

### 2.6. Bactericidal Effect of CSCu or CSZn Hydrogels

**Figure 7:**
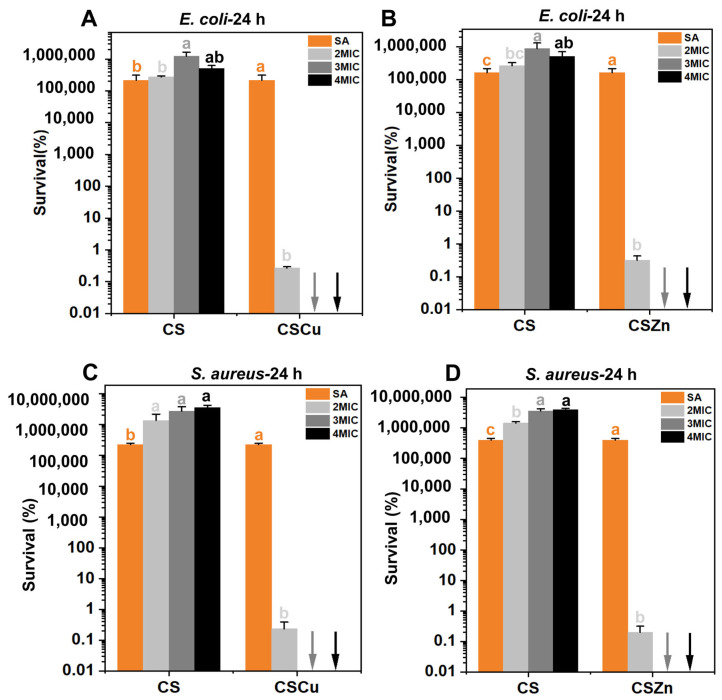
Bactericidal efficacy of CS-, CSCu-, and CSZn-loaded hydrogels against *E. coli* and *S. aureus*. The survival of *E. coli* exposed to CS, CSCu (**A**), or CSZn (**B**) hydrogels. The survival of *S. aureus* exposed to CS, CSCu (**C**), or CSZn (**D**) hydrogels (2MIC, 3MIC, and 4MIC). SA indicates the blank hydrogel without CS, CSCu, or CSZn. All data are expressed as mean ± SD (*n* = 3). Different letters above the bars denote statistically significant differences within the same treatment group (*p* < 0.001) (after taking the logarithm of survival (%), one-way ANOVA analysis was performed). Arrows indicate that no bacterial colonies were observed on the plates.

While the antibacterial activities of CSCu and CSZn complexes in solution were established, the bactericidal efficacy of CSCu or CSZn formulated as a hydrogel required validation to confirm maintenance of antimicrobial function within the matrix. Hydrogel formulations containing 2MIC, 3MIC, and 4MIC CSCu or CSZn were incubated with *E. coli* or *S. aureus* for 24 h, and bacterial survival was quantified (Figure 7). In *E. coli* cultures (Figure 7A,B), both pure SA hydrogels and native CS hydrogels (containing LB medium) seem to support bacterial proliferation under growth conditions. In stark contrast, CSCu and CSZn hydrogels at 3MIC and 4MIC concentrations achieved complete bacterial elimination, with no detectable CFUs recovered. Similarly, against *S. aureus* (Figure 7C,D), SA and CS hydrogels (containing TSB medium) exhibited no antimicrobial effects, while CSCu and CSZn hydrogels at 3MIC and 4MIC achieved total bactericidal elimination. Even at the concentration of 2MIC, the bactericidal activity of CSCu or CSZn hydrogels can exceed 99%.

These results demonstrate that CSCu or CSZn retain potent bactericidal activity when incorporated into hydrogel matrices, with a 3MIC CSCu or CSZn concentration sufficient to achieve complete bacterial killing. The sustained antimicrobial efficacy within the hydrogel matrix indicates controlled release of copper or zinc ions from CSCu or CSZn, enabling prolonged antibacterial action at the wound site. The superior bactericidal performance of CSCu and CSZn hydrogels compared to previously reported CSFe hydrogels [19] further underscores the enhanced antimicrobial potential of copper- or zinc-based polysaccharide complexes, which is likely attributable to the intrinsically stronger antibacterial efficacy of copper and zinc ions [38]. The antibacterial performance of our CSCu and CSZn hydrogels compares similarly with recently reported metal–polysaccharide systems. Suner et al. prepared physically crosslinked CS-Cu(II) and CS-Zn(II) particles with MIC values of 2.5–5.0 mg/mL against *E. coli* and *S. aureus* [39]. Our CSCu hydrogel achieved comparable antibacterial activity against both strains, indicating that alginate incorporation hardly compromises the intrinsic antibacterial activity of the CS–metal complexes. Wu et al. reported CSZn with dual antibacterial and anti-inflammatory functionalities conducive to wound repair [40]. Our work extends this finding by demonstrating that both CSZn and CSCu can be formulated into an injectable, spreadable hydrogel with maintained bioactivity. Furthermore, a recent zinc alginate hydrogel-coated wound dressing achieved a 90.75% healing rate by day 21 in a rat burn model, whereas our CSZn hydrogel achieved 98.11% healing by day 10, suggesting comparable or superior efficacy with accelerated healing kinetics. Notably, Zhao et al. demonstrated that Cu^2+^ induce ferroptosis-like death in *S. aureus* through membrane damage, ROS generation, and iron dysregulation and developed a CuSO_4_-loaded SA hydrogel (Cu-SA) that significantly promoted MRSA-infected wound healing [32]. Given the structural and compositional similarities between their Cu-SA system and our CSCu hydrogel, this study provides strong literature support for the proposed antibacterial mechanism and in vivo efficacy of our CSCu hydrogel. These comparisons collectively underscore the translational potential of our CSCu or CSZn hydrogel system.

While the antibacterial activity of CSCu and CSZn hydrogels was demonstrated against single-species cultures of *E. coli* and *S. aureus*, chronic non-healing wounds are frequently characterized by polymicrobial infections involving multiple bacterial species. Notably, copper and zinc ions are both recognized for their broad-spectrum antibacterial effects, with activity spanning clinically significant wound isolates such as *E. coli*, *S. aureus*, and *P. aeruginosa* (a particularly problematic Gram-negative pathogen commonly found in chronic wounds) [41]. Furthermore, Cu or Zn bimetallic systems have demonstrated synergistic antibacterial effects against multiple species, with enhanced activity against *P. aeruginosa* when copper and zinc are combined [42], and they have been shown to effectively heal mixed bacterial-induced wound infections in vivo [43]. Additionally, copper ions possess potent biofilm-disruption capabilities, and Cu- or Zn-containing biomaterials effectively inhibit bacteria in both planktonic and biofilm forms, and this is a critical feature given that polymicrobial infections in chronic wounds typically exist as complex biofilms refractory to conventional antibiotics. These literature findings strongly suggest that our CSCu or CSZn hydrogel, which releases Cu^2+^ or Zn^2+^ ions, would be effective against mixed-species wound infections. Nevertheless, we acknowledge that direct experimental validation using polymicrobial infection models (e.g., *S. aureus* + *P. aeruginosa* co-culture or mixed-infection wound models) represents an important direction for our future work to fully establish the clinical applicability of these hydrogels for chronic wound management.

### 2.7. Effects of CSCu or CSZn Hydrogel on Wound-Infected Healing

**Figure 8:**
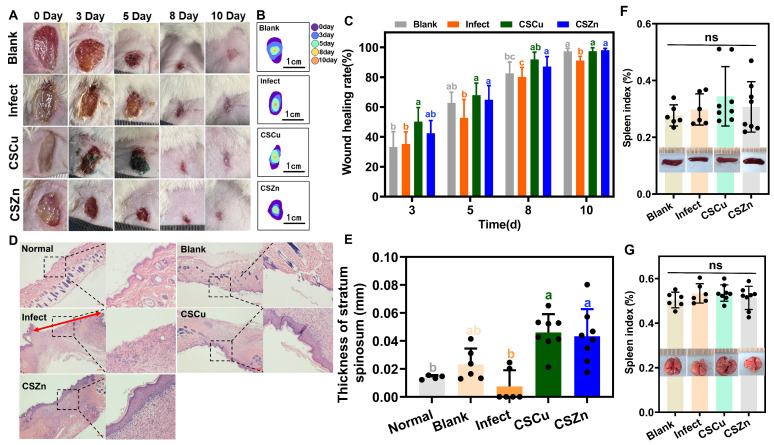
Effects of CSCu and CSZn hydrogels on infected wound healing. (**A**) Photographic images of wounds captured on days 0, 3, 5, 8, and 10 post-treatment. (**B**) Wound areas quantified via ImageJ (ImageJ 1.53a, Wayne Rashband National Institute of Health, Bethesda, MD, USA). (**C**) Healing rates among different groups calculated at the same time points. Values are means ± SD (*n* = 6 or 8 per group). Distinct letters above the bars denote significant differences at *p* < 0.05 (one-way ANOVA, Duncan’s test). (**D**) H&E staining of wound tissue. The red arrow represents unhealed wound tissue. Scale = 100 μm. (**E**) Stratum spinosum thickness measurements (different letters indicate *p* < 0.01, one-way ANOVA, Duncan’s test). Spleen (**F**) and lung (**G**) indices for all four groups. “ns” indicates non-significant differences (*p* > 0.05).

The therapeutic potential of CSCu or CSZn hydrogels was evaluated in a murine model of *S. aureus*-infected full-thickness wounds. Mice were randomized into three groups: blank (non-infected wound), infection (*S. aureus*-infected, untreated), and CSCu or CSZn *(S. aureus*-infected or CSCu or CSZn hydrogel-treated). Wound healing progression was monitored over 10 days through digital photography and quantitative image analysis (Figure 8). As shown in Figure 8A–C, both CSCu and CSZn hydrogels effectively accelerated the healing of infected wounds in mice, as evidenced by the wound images and the quantitative healing rates recorded on days 0, 3, 5, 8, and 10. By day 3, the CSCu hydrogel group exhibited 50.23% wound closure, significantly exceeding both the infection group (35.37%, *p* < 0.05) and the blank group (33.26%, *p* < 0.05). However, there is no difference among the CSZn hydrogel group, infection group, and blank group (*p* > 0.05), and until day 5, the CSCu hydrogel group and the CSZn hydrogel group exhibited good wound-infected healing effects. This early acceleration in healing suggests that CSCu or CSZn hydrogels not only prevent bacterial proliferation but also actively promote the initiation of wound repair processes. By day 8, wound closure in the CSCu group reached 91.87%, and that in the CSZn group reached 87.17%, and both significantly (*p* < 0.05) surpassed the infection group (80.18%) and the blank group (82.54%). At day 10, the CSCu group achieved 97.46% healing, and that in the CSZn group achieved 98.11%, statistically (*p* > 0.05) comparable to the blank group (97.36%) and significantly (*p* < 0.05) superior to the infection group (91.14%). Notably, persistent scab formation remained evident in the infection group at day 10, indicating delayed healing.

Histopathological evaluation of wound tissue by H&E staining revealed distinct tissue architecture among groups (Figure 8D). Normal skin morphology was characterized by intact epidermal stratification including stratum corneum, granulosum, spinosum, and basale layers. In contrast, wound sites from all experimental groups exhibited disrupted architecture. The blank, CSCu, and CSZn groups demonstrated complete wound closure with re-established epidermal continuity, while the infection group showed incomplete healing with persistent inflammatory infiltrate. Quantitative analysis of epidermal thickness revealed that the stratum spinosum was significantly (*p* < 0.05) thicker in the CSCu or CSZn hydrogel group compared to all other groups (Figure 8E). The stratum spinosum, comprising multiple layers of keratinocytes, plays a critical role in epidermal barrier function and wound tensile strength [44]. The enhanced stratum spinosum formation in CSCu- or CSZn-treated wounds suggests accelerated keratinocyte proliferation and differentiation, contributing to improved wound quality and barrier restoration. Assessment of systemic effects through visceral organ indices revealed no significant differences in spleen or lung indices among groups (Figure 8F,G), indicating that topical CSCu or CSZn hydrogel application did not induce systemic toxicity or organomegaly. This favorable safety profile supports the clinical translatability of CSCu and CSZn hydrogels for wound care applications.

Although direct in vitro cytotoxicity assays, together with ion release kinetics, on the hydrogel extracts were not performed in the present study, the biosafety of the CSCu and CSZn complexes is strongly supported by both our in vivo findings and independent literature evidence as shown above. A recent study by Suner et al. demonstrated that CS-Cu(II) and CS-Zn(II) particles exhibited excellent blood compatibility with negligible cytotoxicity toward L929 fibroblasts, even at concentrations up to 10 mg/mL [39], and they showed negligible hemolytic activity, confirming the inherent safety of these CS–metal complexes. While the 10-day in vivo study demonstrated the absence of systemic toxicity, as evidenced by normal spleen and lung indices (Figure 8F,G), the potential for long-term accumulation of Cu^2+^ and Zn^2+^ ions in filtering organs, such as the liver and kidneys, warrants consideration for chronic wound applications. Several factors mitigate this concern. First, the metal ions are chelated to the CS backbone, ensuring sustained release at the wound site and limiting the bolus release that could lead to systemic overload. Second, both copper and zinc are essential trace elements subject to tight homeostatic regulation; notably, chronic non-healing wounds are associated with significantly reduced serum levels of these ions, suggesting that topical supplementation may be physiologically beneficial rather than harmful. Third, the skin provides a substantial barrier to systemic absorption of topically applied metal ions. We acknowledge, however, that direct evidence of long-term biosafety is required for clinical translation. Future studies will systematically evaluate the ion release kinetics and the biodistribution of Cu^2+^ and Zn^2+^ in major organs using ICP-MS analysis after extended hydrogel application in chronic wound models.

**Figure 9:**
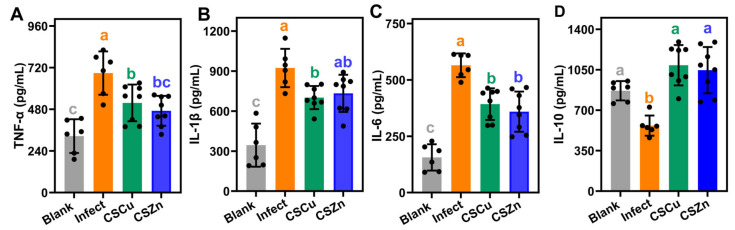
Comparative analysis of serum inflammatory factor levels across the blank, infection, CSCu, and CSZn treatment groups. Levels of the pro-inflammatory cytokines TNF-α (**A**), IL-1β (**B**), and IL-6 (**C**) were measured, alongside the anti-inflammatory cytokine IL-10 (**D**). Data are expressed as mean ± SD (*n*= 6 or 8 per group). Intergroup comparisons were evaluated by one-way ANOVA with Duncan’s test, and distinct letter designations reflect statistical significance at *p* < 0.01.

*S. aureus* is recognized as the predominant pathogen in wound infections, with its remarkable adaptability and capacity for antimicrobial resistance acquisition contributing to persistent infection and delayed healing [45]. *S. aureus* infection triggers inflammatory cascades through activation of pattern recognition receptors, leading to production of pro-inflammatory cytokines including TNF-α, IL-1β, and IL-6 [46]. These cytokines, predominantly derived from M1 macrophages, perpetuate inflammatory states that impair wound healing progression. Conversely, M2 macrophages produce anti-inflammatory IL10, which promotes resolution of inflammation and transition to the proliferative phase of wound repair [47]. To investigate the immunomodulatory effects of CSCu or CSZn hydrogel treatment, serum cytokine levels were quantified at day 10 (Figure 9). The infection group showed marked elevations in TNF-α, IL-1β, and IL-6 compared with the blank group (*p* < 0.05), which confirmed the establishment of a systemic inflammatory response upon S. aureus infection. CSCu or CSZn hydrogel treatment significantly (*p*< 0.01) reduced the concentrations of these pro-inflammatory mediators, indicating effective attenuation of infection-induced inflammation. Concurrently, the anti-inflammatory cytokine IL-10 was significantly (*p* < 0.01) elevated in the CSCu and CSZn group compared to the infection group, suggesting a shift toward an anti-inflammatory, pro-resolution phenotype.

These immunomodulatory effects likely contribute substantially to the accelerated wound healing observed with CSCu or CSZn hydrogel treatment. By reducing pro-inflammatory cytokine burden and enhancing IL-10 production, CSCu or CSZn hydrogels promote a favorable microenvironment for tissue regeneration. The dual mechanism of action—direct antibacterial activity against *S. aureus* combined with modulation of host inflammatory responses—pushes CSCu or CSZn hydrogels as a multifunctional therapeutic platform for infected wound management. Here, it should be noted that the serum cytokine levels were measured only at the endpoint of the 10-day observation period, and, therefore, the early (days 1–3) and mid-phase (days 5–7) cytokine dynamics were not captured in this study. This represents a limitation of our current experimental design. Given that the CSCu or CSZn hydrogel-treated group demonstrated significantly accelerated wound closure beginning as early as day 3 or day 5 and achieving nearly complete healing by day 10 (Figure 8C), it is reasonable to infer that the antibacterial and immunomodulatory actions of the hydrogels effectively curtailed the early inflammatory burst and facilitated the transition to the proliferative phase during the mid-phase of treatment. Nevertheless, we acknowledge that direct temporal profiling would provide more definitive mechanistic evidence. Future studies will incorporate multi-time point cytokine measurements (days 1, 3, 5, 7, and 10) to fully elucidate the dynamic immunomodulatory effects of CSCu or CSZn hydrogel.

Beyond the direct antibacterial and immunomodulatory effects demonstrated above, the accelerated wound healing observed with CSCu or CSZn hydrogel treatment may also involve pro-angiogenic mechanisms mediated by the released Cu^2+^ and Zn^2+^ ions. Copper ions are recognized for their potent capacity to upregulate vascular endothelial growth factor (VEGF) expression in human keratinocytes and, when applied topically, to accelerate wound closure via enhanced angiogenesis [48]. Copper-containing hydrogels exhibit antimicrobial, anti-inflammatory, and angiogenic properties, promoting tissue regeneration through cell adhesion, proliferation, and differentiation [49]. Notably, recent studies have demonstrated that chondroitin sulfate-based biomaterials incorporating metal ions, including Cu^2+^ and Zn^2+^, can effectively promote neovascularization and collagen deposition in wound healing models [50,51]. Although direct histological evidence of neovascularization (e.g., CD31 immunohistochemistry) was not obtained in the present study, the significantly enhanced wound closure rates and improved tissue architecture observed in CSCu- or CSZn-treated groups are consistent with a multifaceted healing mechanism that likely includes angiogenesis, re-epithelialization, and immunomodulation. Future studies will systematically evaluate the pro-angiogenic contribution of these hydrogels through CD31/α-SMA staining and VEGF expression analysis.

## 3. Conclusions

This study successfully fabricated a chondroitin sulfate–metal complex (CSCu and CSZn) through ion exchange methodology. Comprehensive physicochemical characterization confirmed the formation of a structurally stable polysaccharide–metal complex, with Cu^2+^ and Zn^2+^ coordinating to carboxyl, sulfate, or N-acetyl groups of CS. CSCu and CSZn exhibited strong antibacterial effects on *E. coli* and *S. aureus*, with bactericidal efficacy superior to that of native CS and the previously reported chondroitin sulfate–iron complex.

The integration of CSCu or CSZn with SA produced hydrogel formulations exhibiting enhanced rheological properties, injectability, and a highly porous three-dimensional architecture favorable for wound healing applications. In vivo evaluation in a murine model of *S. aureus*-infected wounds demonstrated that topical CSCu or CSZn hydrogel treatment significantly accelerated wound closure, achieving healing rates comparable to non-infected controls by day 10. The therapeutic benefits of CSCu or CSZn hydrogels were attributed to a dual mechanism: a direct bactericidal effect that eradicates the causative pathogen, and an immunomodulatory function reflected by reduced levels of pro-inflammatory mediators TNF-α, IL-1β, and IL-6 and elevated levels of anti-inflammatory IL-10.

Several limitations of this study should be acknowledged. The in vitro cytotoxicity, long-term storage stability, ion release kinetics, swelling ratio, degradation behavior, and quantitative mechanical properties of the hydrogel formulations were not systematically evaluated. Quantitative adhesion and injectability parameters remain to be determined, and a positive control group (commercial wound dressing) was not included in the in vivo study. The in vivo observation was limited to a 10-day period with cytokine profiling only at the endpoint, leaving the early- to mid-phase immune dynamics and potential metal ion accumulation in filtering organs unexplored. Furthermore, the antibacterial efficacy was assessed against single-species cultures rather than polymicrobial infections, and a formal cost-effectiveness comparison with commercial silver-based dressings was not performed. These aspects represent important directions for our future investigations to facilitate the clinical translation of CSCu or CSZn hydrogels. Despite these limitations, our findings establish CSCu and CSZn hydrogels as promising candidates for advanced wound dressings targeting bacterial infections. This work contributes to the expanding repertoire of polysaccharide–metal biomaterials and offers a valuable alternative strategy for addressing the global challenge of antimicrobial resistance in wound care.

## 4. Materials and Methods

### 4.1. Materials

CS derived from bovine cartilage was supplied by the Institute of Food Science and Technology, Chinese Academy of Agricultural Sciences (Beijing, China). Copper (II) chloride dihydrate (CuCl_2_·2H_2_O, purity ≥99%) and zinc chloride (ZnCl_2_) were procured from Tianjin Jinhuitaiya Chemical Reagents Co., Ltd. (Tianjin, China). Sodium alginate (SA, Catalog No. S817374) was obtained from Shanghai Macklin Biochemical Co., Ltd. (Shanghai, China). *S. aureus* (ATCC 25923) and *E. coli* (MG1655) were generously provided by the Shaanxi University of Science and Technology (Xi’an, China). All additional reagents were of analytical grade and utilized without further purification.

### 4.2. Preparation and Physicochemical Characterization of CSCu and CSZn

#### 4.2.1. Preparation of CSCu and CSZn

CSCu and CSZn were fabricated following a modified ion exchange methodology previously described for polysaccharide–metal complex formation [20]. Briefly, CS was dissolved in deionized water under continuous magnetic stirring at ambient temperature (25 °C) to obtain a final concentration of 2.5% (*w*/*v*). Subsequently, CuCl_2_ or ZnCl_2_ solution was added dropwise over a period of 30 min under gentle agitation, maintaining a molar ratio of CS (g) to CuCl_2_·2H_2_O or ZnCl_2_ (mmol) of 1:0.25. The reaction mixture was then stirred continuously at ambient temperature (25 °C) for 12 h to reach equilibrium.

The resulting blue-colored solution underwent purification through three cycles of ethanol precipitation (1:3, *v*/*v*), each followed by centrifugation at 2000× *g* for 2 min. The precipitate was redissolved in deionized water, and this washing sequence was repeated until the supernatant became colorless, indicating complete removal of unbound copper ions. The collected precipitate was redissolved in deionized water, followed by dialysis against distilled water at 4 °C for 4 d through 10 kDa membranes, with fresh dialysate being replaced every 12 h. Finally, light blue CSCu powder and white CSZn powder were obtained after lyophilization of the retentate. The products were then stored in a desiccator for later use.

#### 4.2.2. Microstructure and Elemental Analysis

The microstructure and elemental composition of CSCu, CSZn, and native CS were analyzed by SEM coupled with EDS. Briefly, lyophilized sample powders were mounted onto carbon adhesive conductive tape, and excess materials were gently removed using a nitrogen stream. After gold sputter coating to enhance surface conductivity, the specimens were examined using a COXEM-30 plus SEM (Daejeon, Republic of Korea) equipped with an OXFORD IE250 EDS detector (Oxford, UK) at an accelerating voltage of 20 kV under magnifications of 12,000× and 20,000×.

#### 4.2.3. Molecular Weight Determination

Chromatographic analysis was conducted on an Agilent 1260 Infinity II system (Agilent Technologies, Inc., Waldbronn, Germany) fitted with a TSKgel GMPWXL column and BI-MwA multi-angle light scattering plus differential refractive index detectors. The mobile phase (0.1 M NaCl) ran at 0.5 mL/min, and the column oven was set to 35 °C. Samples were dissolved at 1 mg/mL, syringe-filtered (0.45 μm PVDF), and injected (10 μL). Detector calibration relied on a dextran standard of 102,000 g/mol. This setup was used to determine the Mw values of CSCu, CSZn, and CS.

#### 4.2.4. Metal Content and Surface Charge Analysis

After microwave-assisted digestion, the Cu and Zn levels in CSCu, CSZn, and CS were measured by ICP-MS (7700×, Agilent Technologies Inc., Palo Alto, CA, USA). An autosampler facilitated sample introduction. The surface charge (zeta potential) of CSCu, CSZn, and CS solutions (2.5 mg/mL) was measured at ambient temperature using a Nano9200 zeta potential analyzer (HAIXINRUI, Beijing, China).

#### 4.2.5. Spectroscopic and Structural Analysis

Fourier-transform infrared (FTIR) spectra were acquired on a Nicolet Summit X spectrometer (Thermo Fisher Scientific, Madison, WI, USA) to elucidate coordination interactions between CS and Cu^2+^ or Zn^2+^. Approximately 10 mg of lyophilized CS, CSCu, or CSZn sample powder was mixed with KBr at a 1:200 ratio and compressed into pellets (∼1 mm thickness). Spectra were collected across the 4000–400 cm^−1^ region at 4 cm^−1^ resolution, using air as the background.

XRD measurements were conducted on a D8 Advance ECO diffractometer (XRD, D8 Advance ECO, Bruker, Karlsruhe, Germany) under 30 kV and 10 mA, with diffractograms acquired over a 2θ interval of 10–90°. ^1^H-NMR spectroscopic analysis was performed on a Bruker AMX 400 MHz spectrometer (Bruker, Billerbach, Germany), for which 50 mg of CS, CSCu, or CSZn were each dissolved in 1 mL of D_2_O and transferred to NMR tubes prior to analysis. Spectral data were processed using MestReNova 14.2 software (Mestrelab Research, Santiago de Compostela, Spain).

### 4.3. Antibacterial Activity of CSCu and CSZn Assessment

Antibacterial activity of CSCu, CSZn, and native CS against *E. coli* and *S. aureus* was assessed via the broth microdilution assay in 96-well plates following a published protocol [52]. Specifically, bacterial suspensions (~10^6^ CFU/mL) were treated with CSCu, CSZn, or CS at concentrations of 0, 1.25, 2.5, and 5 mg/mL in Luria–Bertani (LB) medium (for *E. coli*) or Trypticase Soy Broth (TSB) (for *S. aureus*) at 37 °C for up to 72 h. Growth was tracked by OD600 readings using a spectrophotometer. The MIC was determined based on OD600 values and visual assessment of culture medium turbidity.

Following OD600 determination at 72 h, viable bacterial enumeration was performed using the microdilution drop plate method. Specifically, eight serial dilutions of each concentration of CSCu, CSZn, or CS were prepared, and 10 μL of dilution was taken to be spotted onto LB or TSB agar plates, followed by incubation at 37 °C for approximately 12 h. Colony-forming units (CFUs) were quantified to assess the bactericidal activity of CSCu, CSZn, or CS on *E. coli* or *S. aureus*.

### 4.4. CSCu and CSZn Hydrogel Formulation and Characterization

#### 4.4.1. Preparation of CSCu- or CSZn-Loaded Hydrogels

CSCu or CSZn hydrogels were prepared following established protocols with modifications [19]. CSCu or CSZn solutions at concentrations corresponding to 0, MIC, 2MIC, and 3MIC were prepared, with native CS solutions at equivalent concentrations as controls. To obtain CSCu and CSZn hydrogels with final SA concentrations of 1% and 1.5% (*w*/*v*), respectively, SA was incorporated into the solutions under vigorous agitation until complete dissolution and transparency were observed. The resulting mixtures were degassed under vacuum to eliminate air bubbles. Prepared hydrogels were stored at 4 °C until further characterization. Prior to use, all hydrogel formulations were visually inspected for homogeneity and physical integrity. No significant changes in color, transparency, or gel consistency were observed for up to 72 h of storage at 4 °C, indicating adequate short-term physical stability for the subsequent experiments.

#### 4.4.2. Viscosity and Flowability of CSCu or CSZn Hydrogels

The viscosity of CSCu, CSZn, or CS hydrogels was determined by a RH20 rheometer (BosinTech, Shanghai, China) at 25 °C with constant temperature circulating water. Measurements employed a CP50 cone-plate rotor (50 mm diameter, 1° cone angle), and the distance between the platform and rotor was set as 1 mm. The shear rates ranged from 1 to 1000 s^−1^.

Flowability was assessed by tilting 20 mL hydrogel samples (containing 0, 2MIC, and 3MIC of CSCu or CSZn) in 50 mL centrifuge tubes at a 20° angle. Injectability was evaluated by loading 2 mL of CSCu or CSZn hydrogels (3MIC) into a 10 mL medical syringe fitted with a 20G needle. The hydrogel was then manually extruded onto a plastic dish at room temperature (25 °C). Photographs were taken immediately after extrusion and again after 30 min to assess shape retention and structural integrity.

Spreadability was assessed by applying and smearing the hydrogel onto human skin and imaging. To qualitatively assess adhesion under dynamic mechanical stress, approximately 0.5 mL of CSCu or CSZn hydrogels (3MIC) was applied onto the dorsal hand skin and knuckle joint of a healthy volunteer. The volunteer was instructed to perform repeated flexion and extension movements of the fingers at a frequency of approximately 1 cycle per 2 s for about 1 min. The hydrogel behavior was recorded using a digital camera from a fixed distance of 20 cm under ambient light. The full recording was submitted as Supplementary Videos S1 and S2.

#### 4.4.3. Internal Microstructure of CSCu and CSZn Hydrogels

The internal morphological structure of freeze-dried CSCu, CSZn, and CS hydrogels (3MIC) was observed using a ZEISS GeminiSEM 360 (Oberkochen, Germany). Cross-sectional specimens of the freeze-dried hydrogels were prepared using a surgical scalpel, coated with a gold layer, and then observed at 2 kV under 60×, 100×, and 200× magnifications.

### 4.5. Bactericidal Efficacy of CSCu or CSZn Hydrogels

The bactericidal performance of CSCu or CSZn hydrogel formulations was assessed following established procedures [19]. Hydrogel formulations (1 mL) containing SA and about 10^6^ CFU/mL of bacterial cells, *E. coli*, or *S. aureus*, with or without CSCu or CSZn at 2MIC, 3MIC, or 4MIC, were prepared. CS-containing hydrogels at equivalent concentrations served as controls. After 24 h of shaking incubation (37 °C, 200 rpm), sterile saline (about 1 mL) was introduced into each hydrogel tube and mixed, and the aqueous fraction was harvested for CFU enumeration by drop plating (see Section 4.3). Survival rates were then computed via Equation (1):

(1) $$Survival\;ratio\;\left(\%\right)=\frac{{CFU}_{t}}{{CFU}_{0}}\;\times 100$$

where *CFU*_t_ represents the number of CFUs after treatment with CSCu, CSZn, or CS hydrogels, and *CFU*_0_ is the initial bacterial inoculum (~10^6^ CFUs).

### 4.6. In Vivo Wound Healing Study

#### 4.6.1. Animals and Experimental Design

Male SPF KM mice (5 weeks old) were supplied by Henan Skobes Biotechnology Co., Ltd. (Henan, Anyang, China). They were kept in pairs within an SPF barrier facility under controlled environmental conditions (12 h light/dark cycles, 50–70% humidity) with unrestricted access to feed and water. All experimental procedures were approved by the Ethics Committee of Nanyang Institute of Technology (approval No. 2025002) and were conducted in accordance with European Community guidelines (2010/63/EU) for laboratory animal care. Following a one-week acclimatization period, mice were randomly allocated to four groups (n = 28 total): blank group (n = 6, wound surgery only), infection group (n = 6, wound surgery+ *S. aureus* infection), CSCu group (n = 8, wound surgery+ *S. aureus* infection+ CSCu hydrogel treatment), and CSZn group (n = 8, wound surgery+ *S. aureus* infection+ CSZn hydrogel treatment). These experiments were performed concurrently with those reported in our previous study using the same cohort of male SPF KM mice [19]. In compliance with the 3R principle of reduction, some mice in the blank group (wound surgery only) and infection group (wound surgery+ *S. aureus* infection) were shared between the two studies to minimize the total number of animals used. All surgical procedures, housing conditions, and animal care protocols were identical for both studies.

#### 4.6.2. Surgical Procedures and Treatment Protocol

Wound surgery was performed as previously described with slight modifications [35]. Mice were anesthetized with isoflurane, dorsal hair was shaved, and a full-thickness excisional wound (10 mm in diameter) was made on the mid-back, down to the loose subcutaneous layer. For the infection group, CSCu group, and CSZn group, wounds were inoculated with 100 μL bacterial suspension of *S. aureus* (~10^10^ CFU/mL) for three consecutive days to establish infection. Following the infection period, mice in the CSCu group or CSZn group received daily topical application of CSCu hydrogel or CSZn hydrogel (~0.8 mL), spreading to cover the wound for 10 d. Wound morphology was recorded and photographed daily. At study termination, skin tissue surrounding the wound, blood, lung, and spleen were collected for subsequent analyses.

#### 4.6.3. Sample Collection and Biochemical Analyses

At the conclusion of the treatment period, body weight was recorded, and mice were fasted for 12 h prior to sample collection. Blood was obtained from isoflurane-anesthetized mice by orbital puncture, and serum was separated by centrifugation (2000× *g*, 10 min) and stored at −80 °C. Serum cytokine (TNF-α, IL-1β, IL-6, IL-10) concentrations were quantified using mouse-specific ELISA kits (Catalog No. MU30030, MU30369, MU30044, and MU30055 Bioswamp^®^, Bioswamp Life Science Lab, Wuhan, China). Skin samples excised from the wound margin were fixed in 10% neutral buffered formalin and were paraffin-embedded, microtome-sectioned, and stained with H&E for histology examination. Lung and spleen were excised and weighed. The viscera index was calculated by Equation (2):

(2) $$\mathrm{Viscera}\;\mathrm{index}\;\left(\%\right)=\frac{\;Organ\;weight\;(g)}{Body\;weight\;(g)}\times 100$$

### 4.7. Statistical Analysis

All values are reported as mean ± SD. Multiple-group comparisons were evaluated by one-way ANOVA followed by Duncan’s test, while two-group comparisons were assessed via Student’s *t*-test. SPSS (version 22) was employed for all statistical calculations, with statistical significance defined as *p* < 0.05, *p* < 0.01, and *p* < 0.001.

## Figures and Tables

**Table 1:** ^1^H NMR chemical shifts (ppm) of CS, CSZn, and CSCu ^a^.

	CS		CSZn	
	GlcA	GalNAc	GlcA	GalNAc
H1	4.280	4.359	4.360	4.475
H2	3.173	3.827	3.258	3.904
H3	3.394		3.476	
H4	3.603	3.998	3.680	4.069
4S H4	--	4.559	--	4.638
H5	3.457	3.630	3.536	3.708
H6	--		--	

-- Indicates that no proton signal is detected. ^a^ The ^1^H NMR chemical shifts of CSCu are not listed because the paramagnetic Cu^2+^ ions caused severe line broadening and complete suppression of the polysaccharide proton signals, leaving only the solvent (deuterium oxide) peak detectable. Thus, conventional ^1^H NMR is unsuitable for structural characterization of CSCu.

## Data Availability

The data presented in this study are available upon request from the corresponding author.

## Supplementary Materials

The following supporting information can be downloaded at: https://www.mdpi.com/article/10.3390/gels12070633/s1, Videos S1 and S2. CSCu (Video S1) or CSZn (Video S2) hydrogel was applied to the hand skin and over the knuckle joint of a healthy volunteer. The video clearly demonstrates that the hydrogel adheres stably to the skin surface during joint movement without detachment and maintains its gel integrity, even under repeated bending and stretching cycles.
